# Transactions of the Medical Society of the State of New York for 1888

**Published:** 1888-09

**Authors:** 


**Transactions of the Medical Society of the State of New York for 1888.** Published by the Society. 8vo., pp. 691—12.

The annual Transactions of this ancient and honorable society usually appear somewhat tardily, and this volume is no exception to the rule. Its pages are, however, replete with interesting material, among which may be mentioned the discussion on "Acute Bright's Disease, that on "Intestinal Obstruction," and Dr. Vander Veer's "History of Abdominal Section," in Albany.

An interesting biographical sketch of the late Dr. Thomas F. Rochester, by his son Dr. Delancey Rochester, appears among the obituary memoirs.

It is surprising that a medical society of the age and standing of, and representing in tradition, historic memories, and scientific work all that the Medical Society of the State of New York does, should be content to issue its Transactions from year to year in such an unattractive form, and with such inferior paper and press-work. The pages do not run uniform in color for ink, some being paler than others, and altogether it is not what a first-class printing-office should turn out.

